# A Rare Case of Colonic Triplication with Associated Imperforate Anus in a Newborn Male

**DOI:** 10.1055/s-0042-1750318

**Published:** 2022-08-16

**Authors:** Elise McKenna, Christina Ho, Andrea Badillo, Gustavo Villalona, Marc A. Levitt

**Affiliations:** 1 Department of Colorectal and Pelvic Reconstruction, Surgery, Children's National Hospital, Washington, District of Columbia, United States; 2 Department of Urology, Children's National Hospital, Washington, District of Columbia, United States; 3 Department of Pediatric Surgery, Wolfson Children's Hospital, Jacksonville, Florida, United States

**Keywords:** anorectal malformation, colonic triplication, imperforate anus, rectovesical fistula, colonic duplication

## Abstract

We present a case of a newborn male with imperforate anus who was found to have colonic triplication with a high rectovesical fistula. The case is presented with a focus on surgical strategies for the management of this rare malformation.

## Introduction

Alimentary tract duplications are rare congenital lesions that can occur anywhere from the mouth to the anus. Large bowel duplications account for only a small percentage (∼10%) of all alimentary tract duplications. 1 Triplication of the colon is even rarer, with only a handful of cases described in the literature. Colorectal tubular duplications may be associated with rectogenital or rectourinary fistulae, duplication of internal or external genitalia, or vertebral anomalies. 1 The surgical treatment of these complex hindgut duplications can be very challenging. Here we present a case of a newborn male with colonic triplication and imperforate anus with a high rectovesical fistula.

## Case Presentation

**Fig. 1:**
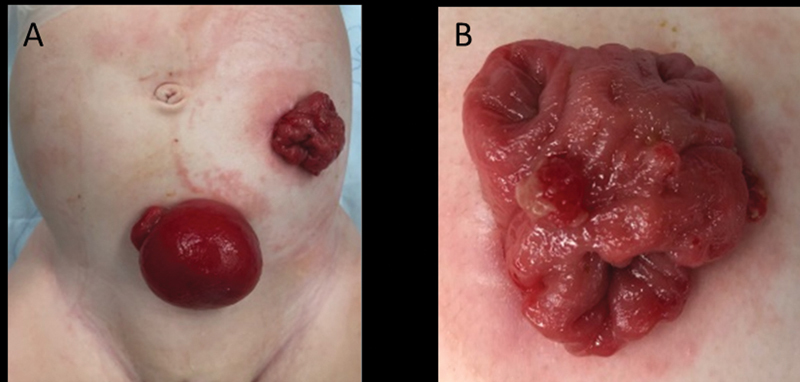
Colostomy and prolapse mucus fistula ( **A** ). Three distinct lumens of the colostomy ( **B** ).

A newborn male, born full-term, weighing 4kg, with no relevant prenatal history, was found to have an anorectal malformation (ARM) in the delivery room. He was taken to the operating room at birth to create a colostomy with mucus fistula. Upon division of the colon, he was noted to have three separate proximal colonic lumens and two distal colonic lumens. The ostomy and mucus fistulas were matured ( Fig. 1 ) and the patient was referred to our center for further management.

**Fig. 2:**
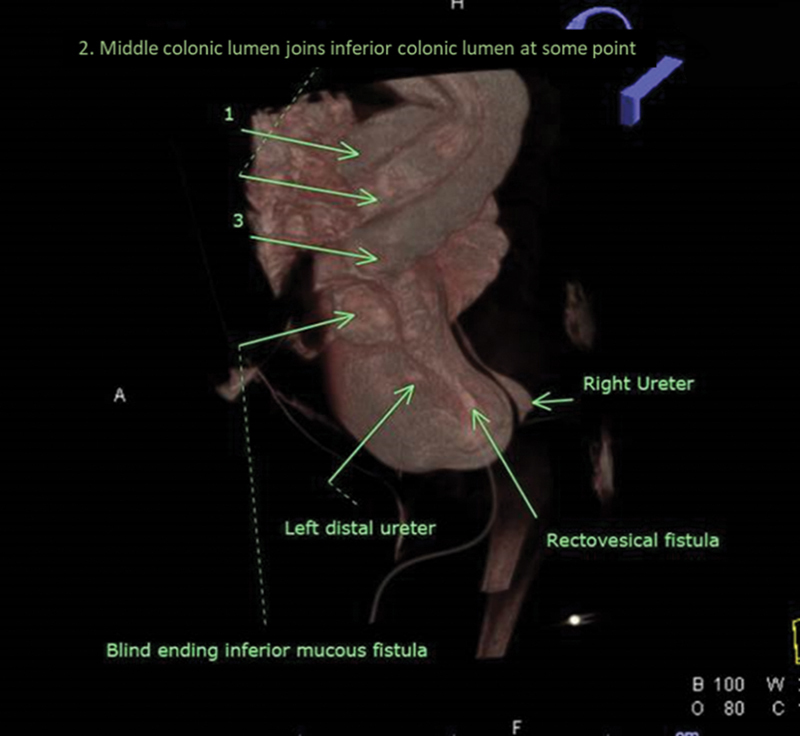
High-resolution three-dimensional imaging showing three colonic lumens of proximal colon, distal colon with one blind ending lumen, and one fistulizing to the bladder.

**Fig. 3:**
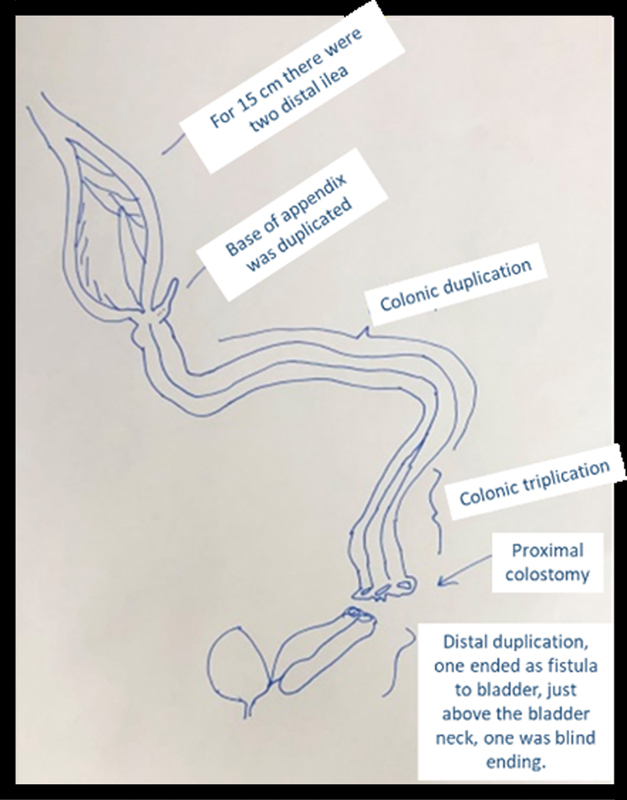
Diagram of the operative findings.

Upon presentation to our center at 5 months of age, an exam under anesthesia, cystoscopy, and contrast studies of the colon were performed. There were three easily identifiable colonic lumens on the end colostomy. The mucus fistula was prolapsed and only two lumens were able to be identified There was no anus identified, but there was a good gluteal cleft. On cystoscopy, a colovesical fistula was seen just distal to the bladder neck, in close proximity to the left ureteral orifice. High-resolution and three-dimensional contrast imaging demonstrated two lumens of the distal colon with one ending as a fistula to the bladder and the other blind ending ( Fig. 2 ). Proximally there were three lumens in the descending colon, which merged to form two lumens around the splenic flexure. The colonic duplication continued all the way to the cecum ( Fig. 3 ).

**Fig. 4:**
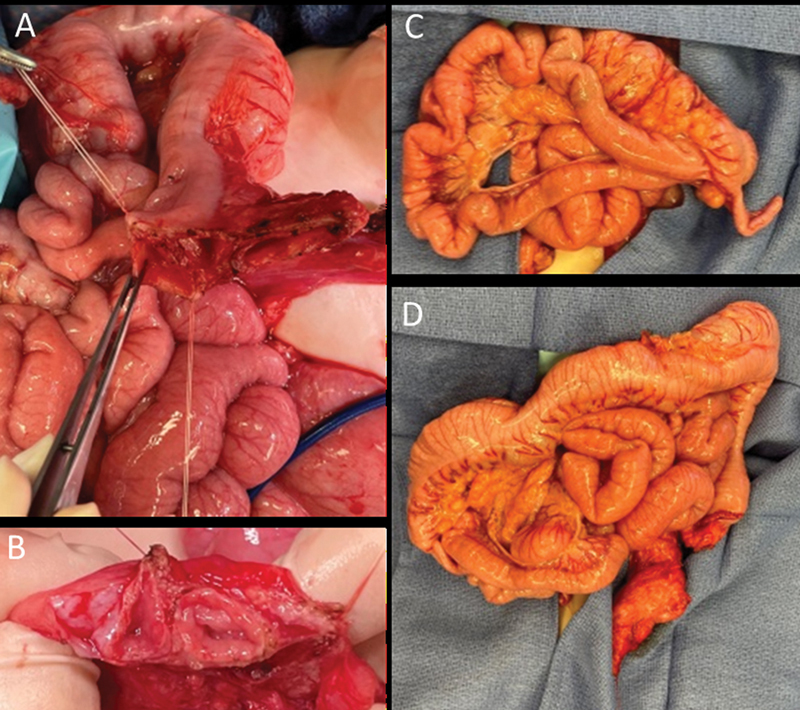
Intraoperative findings. Three colonic lumens of colostomy ( **A** ). Two colonic lumens of mucus fistula ( **B** ). Ileal, cecal, and appendiceal duplication ( **C** ). Entire colon with normal outer appearance despite multiple internal lumens ( **D** ).

The patient was taken to the operating room where an exploratory laparotomy was performed. The anal sphincters were marked at the start of the case using an electrical stimulator. An ileal duplication and fused appendiceal duplication were also identified intraoperatively ( Fig. 4 ). A stapler was used to create a single lumen in the ascending and descending colons ( Fig. 4 ). A posterior sagittal anorectoplasty (PSARP) was performed, the distal rectum connected to the bladder neck was separated, and then the two distal lumens were made into one with stapler and pulled through for the anoplasty. The colostomy was closed, and the thinner of the two distal ileums was resected. A diverting ileostomy was created to protect the repair and was subsequently reversed 6 weeks later. The patient is currently clean with Malone flushes and is working toward control of the flushes as a bridge to voluntary bowel movements.

## Discussion

Triplication of the colon is extremely rare, with only a few cases reported in the literature. 2 3 4 5 These complex patients create a unique surgical challenge for which there is no defined approach. In a patient such as this, diversion is the most appropriate initial step. The anatomy should be well delineated and a comprehensive surgical plan created prior to undertaking major reconstruction.

At reconstruction, creating a single distal rectum with no communication with the urinary tract is vital to success. Any remnant of the original fistula that remains can lead to urinary tract infections, kidney damage, and even affect continence. It is also important to use a muscle stimulator to premark the anal sphincters prior to dissection to place the rectum within the center of the muscle complex.

Another important consideration in the surgical approach to these patients should always include the possibility that they may need management of fecal or urinary incontinence in the future. Using the ARM continence index 6 as a guide, we anticipated that this patient has moderate potential for bowel control because they have a high rectovesical fistula, a normal spine, and an intermediate sacral ratio. The ARM index is a nonvalidated way to guide conversations between clinician and parents of an ARM child, as it appears that prognosis for bowel control is dependent on the type of malformation, and the quality of the sacrum and spine. To account for this, we left the duplicated appendix untouched and created a single lumen channel at the start and end of the colon so that if mechanical flushes are needed, antegrade flushes would achieve emptying of the entire colon at the same time. While we did leave a short area of duplication in the transverse colon, the two lumens were equal in size and communicating, so we do not anticipate any issues with the antegrade flushes he will likely need in the future.

## Conclusion

Colonic triplication with associated imperforate anus and rectovesical fistula is a rare congenital malformation that requires a complex surgical approach. In addition to correction of the imperforate anus and repair of any fistula, one must plan for the future possibility of bowel/bladder incontinence by keeping the appendix for future use and creating a single colonic lumen for mechanical antegrade flushes.

## Notes

New Insights and the Importance for the Pediatric Surgeon Colonic triplication with associated imperforate anus and rectovesical fistula is a rare congenital malformation that requires a complex surgical approach. In addition to correction of the imperforate anus and repair of any fistula, one must plan for the possibility of bowel/bladder incontinence by keeping the appendix for future use and creating a single colonic lumen for mechanical antegrade flushes

## References

[1] Patiño Mayer J Bettolli M Alimentary tract duplications in newborns and children: diagnostic aspects and the role of laparoscopic treatment World J Gastroenterol 2014 20 39 14263 14271 25339813 10.3748/wjg.v20.i39.14263 PMC4202355

[2] Gray A W Triplication of the large intestine Arch Pathol (Chic) 1940 30 1215 1222

[3] Ravitch M M Hind gut duplication; doubling of colon and genital urinary tracts Ann Surg 1953 137 05 588 601 13041109 10.1097/00000658-195305000-00002 PMC1802697

[4] Gisquet H Lemelle J L Lavrand F Droulle P Schmitt M Colonic triplication associated with anorectal malformation: case presentation of a rare embryological disorder J Pediatr Surg 2006 41 07 e17 e19 10.1016/j.jpedsurg.2006.03.016 16818042

[5] Sarin Y K Manchanda V Sharma A Singhal A Triplication of colon with diphallus and complete duplication of bladder and urethra J Pediatr Surg 2006 41 11 1924 1926 17101372 10.1016/j.jpedsurg.2006.06.002

[6] Wood R J Levitt M A Anorectal malformations Clin Colon Rectal Surg 2018 31 02 61 70 29487488 10.1055/s-0037-1609020 PMC5825858

